# Outcomes of Laparoscopic Suture Rectopexy Versus Laparoscopic Mesh Rectopexy: A Systematic Review and Meta-Analysis

**DOI:** 10.7759/cureus.61631

**Published:** 2024-06-04

**Authors:** Meena Kumari, Mangalapalle MadhuBabu, Harsh Vaidya, Kushal Mital, Bharati Pandya

**Affiliations:** 1 Department of General Surgery, All India Institute of Medical Sciences, Bhopal, IND; 2 Department of Surgery, All India Institute of Medical Sciences, Bhopal, IND; 3 Department of Coloproctology, King Edward Memorial Hospital and Seth Gordhandas Sunderdas Medical College, Mumbai, IND

**Keywords:** clinical outcomes, postoperative, full-thickness rectal prolapse, complete rectal prolapse, laparoscopic rectopexy, mesh rectopexy, suture rectopexy

## Abstract

The contemporary literature provides conflicting evidence regarding the precedence of laparoscopic mesh rectopexy over laparoscopic suture rectopexy for full-thickness rectal prolapse. This study aimed to compare the clinical outcomes of mesh and suture rectopexy to improve the surgical management of complete rectal prolapse. The Preferred Reporting Items for Systematic Reviews and Meta-Analyses (PRISMA) guidelines were followed to extract studies based on mesh versus suture rectopexy and published from 2001 to 2023. The articles of interest were obtained from PubMed Central, Cumulative Index to Nursing and Allied Health Literature (CINAHL), Journal Storage (JSTOR), Web of Science, Embase, Scopus, and the Cochrane Library. The primary outcomes included rectal prolapse recurrence, constipation improvement, and operative time. The secondary endpoints included the Cleveland Clinic Constipation Score, Cleveland Clinic Incontinence Score, intraoperative bleeding, hospital stay duration, mortality, overall postoperative complications, and surgical site infection. A statistically significant low recurrence of rectal prolapse (odds ratio: 0.41, 95% confidence interval (CI) 0.21-0.80; p=0.009) and longer mean operative duration (mean difference: 27.05, 95% CI 18.86-35.24; p<0.00001) were observed in patients with mesh rectopexy versus suture rectopexy. Both study groups, however, had no significant differences in constipation improvement and all secondary endpoints (all p>0.05). The laparoscopic mesh rectopexy was associated with a low postoperative rectal prolapse recurrence and a longer operative duration compared to laparoscopic suture rectopexy. Prospective randomized controlled trials should further evaluate mesh and suture rectopexy approaches for postoperative outcomes to inform the surgical management of complete rectal prolapse.

## Introduction and background

The rectal procidentia, or full-thickness prolapse of the rectum, is the outcome of pelvic floor dysfunction, leading to the complete expulsion of the rectum through the anal sphincters [1]. The frequent symptoms of a complete rectal prolapse include fecal incontinence and rectal bleeding [2,3]. The anatomic abnormalities associated with a complete rectal prolapse include anterior cul-de-sac extension, diastasis of the levator muscle, and a reduction in anal sphincter tone [4]. The sphincter complex injury or chronic straining, resulting in sphincter weakness, increases the risk and incidence of complete rectal prolapse [1,4]. The potential risk factors for rectal prolapse include elevated intra-abdominal pressure, chronic constipation, perineal injury, obesity, pregnancy, psychiatric disorders, and connective tissue diseases [1].

The prevalence of complete rectal prolapse in the general population is 0.5% [5]. However, a 2.5% annual incidence of rectal prolapse is reported in patients ≥50 years, who often present with tenesmus, pain, rectal bleeding, incomplete evacuation, incontinence, and constipation [5]. Of note, the rates of constipation and fecal incontinence in such patients are 25-50% and 50-75%, respectively [5]. The progression of rectal prolapse is further aggravated by several potential factors, including pelvic floor weakness, insufficient recto-sacral fixation, redundant sigmoid colon, and a deep Douglas pouch [6]. Additionally, the straining chain, constipation, and outlet obstruction are initiated by external sphincter tonus elevation in patients with rectal prolapse [7]. The optimization of surgical approaches for rectal prolapse depends on a range of factors including differential diagnosis, patient presentation, and the incidence of clinical complications including fecal incontinence, diarrhea, constipation, mucus discharge, incomplete bowel evacuation, and abdominal discomfort [5]. The preoperative scoring systems, such as the Wexner Constipation Score, Rome IV Criteria, Altomare Obstructed Defecation Syndrome (ODS) Score, Pescatori Incontinence Score, Fecal Incontinence Severity Index (FISI), and St. Mark's Incontinence Score (SMIS) facilitate the assessment of rectal prolapse complications [8-10].

Suture rectopexy was initially adopted for rectal fixation/redundant bowel plication; however, it has been increasingly replaced with mesh rectopexy to minimize postprocedural morbidity and chronic pain [11]. Surgeons utilize a laparotomy or laparoscopic approach to perform a suture rectopexy, which was initially elaborated in 1959 by Cutait [11]. The suture rectopexy utilizes a non-absorbable suture for rectal fixation/mobilization [12]. This approach facilitates the integration of the presacral fascia with the rectum, by allowing the growth of adhesions and fibrosis, after its mobilization and suturing [13]. Literature evidence reveals better clinical outcomes in males, treated with suture rectopexy, than females because of their undiagnosed preoperative occult sphincter defects [14].

Ripstein initially described the treatment of complete rectal prolapse with mesh rectopexy in 1952 [11]. Of note, mesh rectopexy fixates the rectum through a non-absorbable/absorbable synthetic material (or anterior sling) following its mobilization [14]. The rectal promontory is finally sutured with the mobilized rectum to reinstitute its natural curve [11]. The utilization of rectal rectopexy for the repair of rectal prolapse improves the clinical outcomes by reducing the downward abdominal pressure [11]. The better anatomic positioning of the rectum via mesh rectopexy is due to its ability to rigidly support the anterior fascia through a non-elastic synthetic graft [15]. This is why patients with noticeable pelvic floor descent also benefit from the mesh rectopexy procedure [16]. 

The current literature provides conflicting evidence about the outcomes of laparoscopic suture versus mesh rectopexy in patients with complete rectal prolapse. For example, the meta-analysis by Lobb et al. defies a significant difference in postprocedural rectal prolapse prevalence between suture rectopexy and mesh rectopexy [11]. Alternatively, the observational study by Takahashi et al. reveals reduced occurrences of complete rectal prolapse after laparoscopic suture rectopexy, compared to mesh rectopexy [17]. Contrastingly, the meta-analyses by Hajibandeh et al. and Emile et al. indicate prolonged procedure duration and lower rectal prolapse recurrence rates with laparoscopic mesh rectopexy versus suture rectopexy [18,19]. Similarly, studies provide conflicting results on several other mesh/suture rectopexy parameters, including mortality, postoperative complications, Wexner scores, constipation/incontinence improvement, intraoperative bleeding, and surgical site infection.

This systematic review and meta-analysis accordingly aimed to address and compare current gaps regarding the clinical outcomes of laparoscopic mesh versus suture rectopexy in patients treated for full-thickness rectal prolapse. 

## Review

Study design and data collection

This systematic review and meta-analysis was undertaken in concordance with the Preferred Reporting Items for Systematic Reviews and Meta-Analyses (PRISMA) guidelines [20]. Retrospective, prospective, and single-/double-blind randomized controlled/comparative studies were evaluated for several postprocedural clinical outcomes in patients with laparoscopic posterior mesh rectopexy versus laparoscopic ventral/posterior mesh rectopexy for full-thickness/complete rectal prolapse. The studies published from 2001 to 2023 were included in this analysis. Of note, articles based on meta-analyses, systematic reviews, review papers, narrative reviews, opinion papers, editorials/correspondences, case studies, and case reports were excluded from this study. All data were initially collected on a Microsoft Excel sheet. Double data checks by two independent reviewers ascertained the avoidance of data entry errors.

Primary and secondary outcomes

The primary outcomes were postoperative rectal prolapse recurrence, constipation improvement, and mean operative time (minutes). The secondary outcomes included Cleveland Clinic Constipation Score (CCCS), Cleveland Clinic Incontinence Score (CCIS), intraoperative bleeding, mean hospital stay duration (postoperative days), mean operative time (minutes), mortality, overall postoperative complications, and surgical site infection. 

Literature search

The literature search was conducted on February 29, 2024, by five autonomous reviewers. We explored several databases, including PubMed Central, Cumulative Index to Nursing and Allied Health Literature (CINAHL), Journal Storage (JSTOR), Web of Science, Embase, Scopus, and the Cochrane Library, to identify the published peer-reviewed articles based on the outcomes of laparoscopic mesh versus suture rectopexy for complete rectal prolapse. The following search terms were combined via Boolean operators (OR and NOT) and executed via advanced filters of the selected databases: (1) Rectal prolapse, (2) Full-thickness, (3) Laparoscopic, (4) Mesh rectopexy, and (5) Suture rectopexy. Title and abstract-based searches were undertaken, followed by the assessment of full-text articles. Data were thoroughly evaluated following the eligibility parameters. Discrepancies in literature search and data collection between the two reviewers were resolved with mutual consensus, following the intervention of a third independent reviewer. 

Statistical analysis

The authors of this study utilized RevMan (Web version) to statistically evaluate the primary and secondary clinical outcomes [21]. Odds ratios (ORs), within 95% confidence intervals (CIs), were calculated to determine the occurrence of intraoperative bleeding, mortality, overall operative complications, postoperative constipation improvement, rectal prolapse recurrence, and surgical site infections [22]. Alternatively, mean differences (MDs) with standard deviations (SDs) were calculated for CCCS, CCIS, hospital stay duration, and operative time in both study groups [23]. The heterogeneity among the included studies was determined by tau-squared, chi-squared, and I-squared statistics [24,25]. The minimal, moderate, and high heterogeneity levels were indicated by 0-30%, 31-60%, and 61-100% I-squared values, respectively. The statistical analyses of the dichotomous data type were undertaken via the Mantel-Haenszel random-effects approach [26]. However, continuous data were examined via the inverse variance random-effects model [27]. The statistical significance of outcomes was evaluated via the probability value reference (p≤0.05) [28]. 

Risk of bias (ROB)

The Cochrane risk of bias in non-randomized studies of interventions (ROBINS-I) tool was used to evaluate the ROB for non-randomized/observational studies [29]. Alternatively, the risk-of-bias visualization (ROBVIS) ROB-2 tool was used to evaluate ROB in randomized controlled studies [30]. Accordingly, the traffic light plots and summary plots were obtained for observational and randomized studies, respectively. ROBs were calculated based on a multitude of parameters, including confounding, participant selection, intervention classification, intervention deviation, missing data, outcome measurement, result selection, and randomization. ROBs were further classified/scaled as low risk, moderate risk, critical risk, no information, and some concerns, respectively.

Results

**Figure 1:**
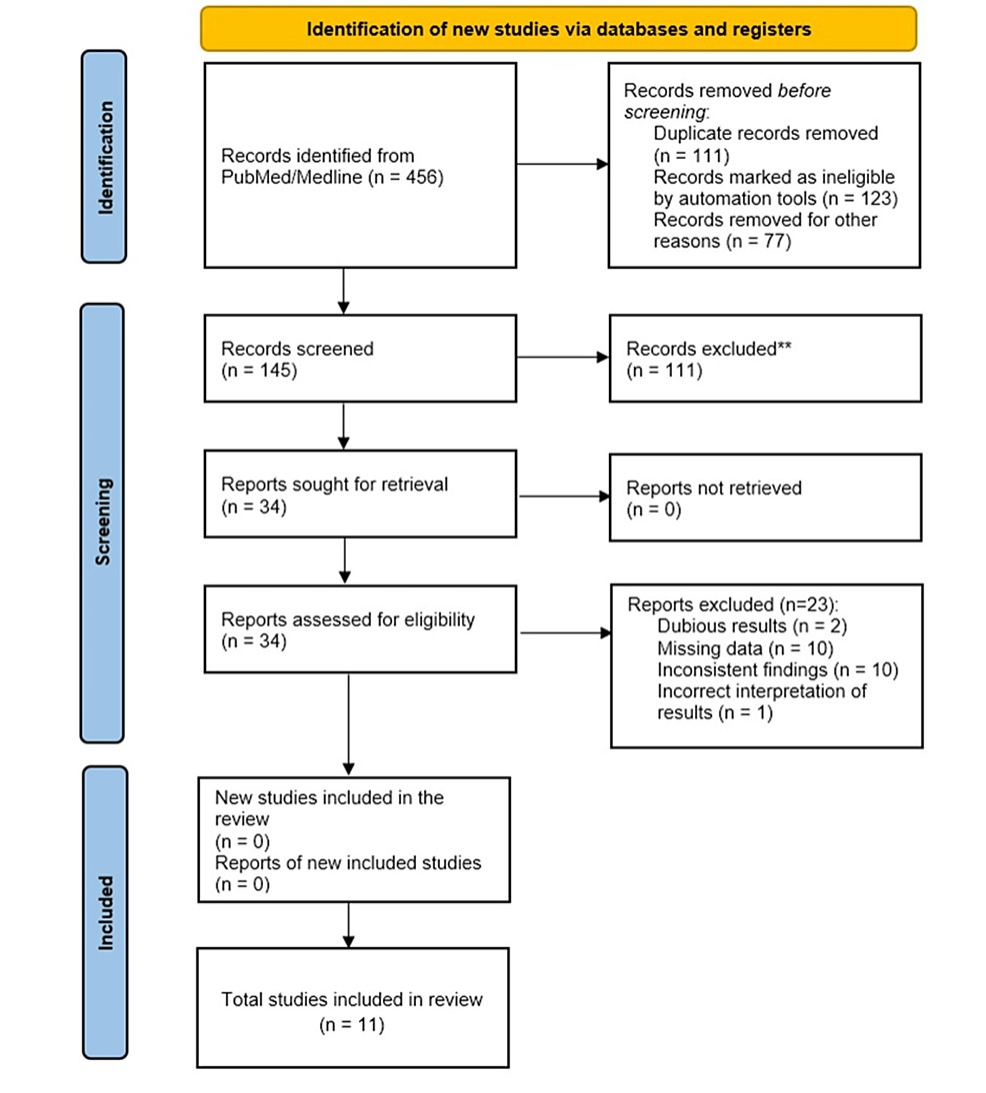
Study flow diagram

The literature search resulted in the selection of 456 studies from PubMed/Medline and CINAHL and 289 studies from Web of Science, JSTOR, and Scopus (Figure 1). One hundred forty-five records were subsequently retrieved after removing the duplicate entries. Of them, 111 records were excluded due to duplicate data. Finally, 34 full-text articles were screened for eligibility, and 11 of them were included for systematic review and meta-analysis after excluding 23 studies based on dubious results, missing data, inconsistent findings, and incorrect interpretation.

Table 1 describes the baseline and preoperative characteristics of participants in each study group. A total of 2362 patients with mesh rectopexy and 2172 patients with suture rectopexy were evaluated for the operative/intraoperative/postoperative outcomes. The mean age of the participants was 55±11 years, and the mean female-to-male ratio was 25/5±0.5. However, most of the included studies did not provide data on preprocedural characteristics, including comorbidities and medication use.

**Table 1:** Baseline and preoperative characteristics ASA: American Society of Anesthesiologists scoring

Characteristics	Gleditsch et al. 2018 [31]		Hidaka et al. 2019 [32]		Benoist et al. 2001 [33]		Luglio et al. 2016 [34]		Lundby et al. 2016 [35]		Mohammedy et al. 2023 [36]		Raftopoulos et al. 2005 [37]		Sahoo et al. 2014 [38]		Usman et al. 2023 [39]		Yehya et al. 2020 [40]		Bhandarwar et al. 2017 [41]	
	Mesh rectopexy	Suture rectopexy	Mesh rectopexy	Suture rectopexy	Mesh rectopexy	Suture rectopexy	Mesh rectopexy	Suture rectopexy	Mesh rectopexy	Suture rectopexy	Mesh rectopexy	Suture rectopexy	Mesh rectopexy	Suture rectopexy	Mesh rectopexy	Suture rectopexy	Mesh rectopexy	Suture rectopexy	Mesh rectopexy	Suture rectopexy	Mesh rectopexy	Suture rectopexy
Patients, n	22	49	34	30	14	16	20	20	38	37	20	20	191	70	38	32	41	34	30	28	25	23
Mean/median age (years)	72 (26-97)	72 (26-97)	56.5 (42-73)	48.5 (33-63)	66.3 (17.3)	76.2 (13)	-	-	60 (44-74)	60 (44-74)	-	-	53 (18-93)	53 (18-93)	42.5 (20-65)		32.5±8.9	41.5±11.7	-	-		25-75
Sex ratio (female/male)	77/16		31/3	27+/3		12/2	16/0	-	35/3	33/4	-	-	-	-	-	-	22/19	19/15	11/19	11/17		-
ASA ≤2	-	-	1 (1-2)	-	7 (50%)	7 (44%)	-	-	37 (48.5%)	-	-	-	-	-	-	-	-	-	-	-	-	-
ASA >2	-	-	-	-	7 (50%)	9 (56%)	-	-	-	-	-	-	-	-	-	-	-	-	-	-	-	-
Body mass index (kg/m²)	-	-	23.1 (21.4-25.9)	-	-	-	-	-	23.4 (21.3-25.7)	-	-	-	-	-	-	-	-	-	-	-	-	-
Prior pelvic surgery	-	-	-	-	3 (21%)	6 (38%)	-	-	-	-	-	-	-	-	-	-	-	-	-	-	-	-
Symptom duration (months)	-	-	-	-	29 (22)	34 (32)	-	-	-	-	-	-	-	-	-	-	-	-	-	-	-	-
Normal continence	-	-	-	-	8 (57%)	8 (57%)	-	-	-	-	-	-	-	-	-	-	-	-	-	-	-	-
Flatus incontinence	-	-	-	-	-	1 (6%)	-	-	-	-	-	-	-	-	-	-	-	-	-	-	-	-
Liquid stool incontinence	-	-	-	-	3 (21.5%)	4 (25%)	-	-	-	-	-	-	-	-	-	-	-	-	-	-	-	-
Solid stool incontinence	-	-	-	-	3 (21.5%)	4 (25%)	-	-	-	-	-	-	-	-	-	-	-	-	-	-	-	-
Severe constipation	-	-	-	-	5 (36%)	6 (38%)	-	-	-	-	-	-	-	-	-	-	-	-	-	-	-	-
Laxative utilization	-	-	-	-	5 (36%)	6 (38%)	-	-	-	-	-	-	-	-	-	-	-	-	-	-	-	-
Aperients utilization	-	-	-	-	3 (21%)	3 (21%)	-	-	-	-	-	-	-	-	-	-	-	-	-	-	-	-
Preoperative Wexner Constipation Score (median, range)	-	-	-	-	-	-	22 (18-26)	21 (17-25)	-	-	9 (8-11)	8.5 (6-14)	-	-	19	18	-	-	-	-	-	-
Preoperative Wexner Incontinence Score (median, range)	-	-	-	-	-	-	12 (8-13)	11 (9-13)	-	-	12 (9-14)	10 (7.75-12.5)	-	-	20	21	-	-	-	-	-	-
Preoperative constipation	-	-	-	-	-	-	-	-	5 (13%)	6 (16%)	-	-	-	-	-	-	-	-	-	-	-	-
Previous surgery (overall)	-	-	-	-	-	-	-	-	17 (45%)	15 (41%)	-	-	-	-	-	-	-	-	-	-	-	-
Previous gynecological surgery	-	-	-	-	-	-	-	-	10/17 (59%)	12/15 (80%)	-	-	-	-	-	-	-	-	-	-	-	-
Previous colonic or rectal surgery	-	-	-	-	-	-	-	-	0/17	0/15	-	-	-	-	-	-	-	-	-	-	-	-
Previous appendectomy	-	-	-	-	-	-	-	-	7/17 (41%)	1/15 (7%)	-	-	-	-	-	-	-	-	-	-	-	-
Previous upper abdominal surgery	-	-	-	-	-	-	-	-	0/17	1/15 (7%)	-	-	-	-	-	-	-	-	-	-	-	-
Previous other surgery	-	-	-	-	-	-	-	-	0/17	1/15 (7%)	-	-	-	-	-	-	-	-	-	-	-	-
Prolapse duration	-	-	-	-	-	-	-	-	-	-	-	-	-	-	-	-	4.7±0.9	4±0.8	-	-	-	-
Prolapse length	-	-	-	-	-	-	-	-	-	-	-	-	-	-	-	-	11.5±3.5	10±3.4	-	-	-	-
Preoperative bleeding per rectum	-	-	-	-	-	-	-	-	-	-	-	-	-	-	-	-	-	-	24	22	-	-
Preoperative rectal pain	-	-	-	-	-	-	-	-	-	-	-	-	-	-	-	-	-	-	3	3	-	-

Table 2 synthesizes details of the authors, publication year, study type, patient classification, study methods, and outcomes. 

**Table 2:** Systematic review ODS: Obstructed Defecation Syndrome

Authors	Publication year	Study type	Patients (n)	Methods/interventions	Results/inferences
Gleditsch et al. [31]	2018	Retrospective study	93 patients, treated with ventral mesh rectopexy or posterior suture rectopexy	Patients were followed up for clinical outcomes for 19 years, after ventral mesh/suture rectopexy for rectal prolapse	No mortality was reported in patients with ventral mesh rectopexy. The recurrence rates for ventral mesh rectopexy and posterior suture rectopexy were 14% and 31%, respectively. Overall complication rates following both procedures were low and did not differ significantly between the study groups
Hidaka et al. [32]	2019	Double-blind, randomized, single-center study	Laparoscopic ventral mesh rectopexy (n=37); laparoscopic posterior sutured rectopexy (n=38)	A six-year follow-up was undertaken for constipation symptom/incontinence scores, mesh complications, and recurrences of rectal prolapse following the successful completion of the suture/mesh rectopexy	At the follow-up of 6.1 years, the Patient Assessment of Constipation Quality of Life was significantly higher in laparoscopic posterior sutured rectopexy patients compared to those with laparoscopic ventral mesh rectopexy. The Cleveland Clinic Constipation Scores favored mesh rectopexy over suture rectopexy. Rectal prolapse recurrence rates were higher in the sutured rectopexy group compared to the mesh rectopexy patients (23.33% vs. 8.82%)
Benoist et al. [33]	2001	Retrospective study	Laparoscopic mesh rectopexy (n=14); laparoscopic suture rectopexy (n=16); resection rectopexy (n=18)	While laparoscopic posterior mesh rectopexy was administered to 14 patients, laparoscopic suture rectopexy, with/without sigmoid resection, was provisioned for 34 patients with full-thickness rectal prolapse	None of the study groups had postoperative mortality. While mucosal prolapse was reported in one patient, a nearly 75% improvement in incontinence rate was observed in all study groups. The highest prevalence of postoperative constipation was observed in patients with mesh rectopexy (64%), followed by those with suture rectopexy (62%) and resection rectopexy (11%). The overall findings did not advocate the superiority of mesh rectopexy over suture rectopexy
Luglio et al. [34]	2016	Prospective study	Laparoscopic suture rectopexy (n=20); laparoscopic mesh rectopexy (n=20)	One-year assessment of clinical outcomes after suture versus mesh rectopexy was undertaken via defecography and recto-sigmoidoscopy	The median postoperative Wexner Constipation Scores were 11 and 15 in mesh and suture rectopexy groups, respectively. The median postoperative incontinence scores in mesh and suture rectopexy groups were 6 and 9, respectively. Higher occurrences of rectal prolapse were observed in patients with suture rectopexy compared to those with mesh rectopexy (n=3 vs. n=1)
Lundby et al. [35]	2016	Randomized, double-blind, single-center study	Laparoscopic ventral mesh rectopexy (n=38); laparoscopic posterior sutured rectopexy (n=37)	The patients were evaluated for ODS Score before and after undergoing sutured/mesh rectopexy	The postoperative ODS Score reductions were 2.18 and 1.97 in sutured rectopexy and mesh rectopexy patients, respectively (p=0.890)
Mohammedy et al. [36]	2023	Prospective, randomized, comparative study	Laparoscopic ventral mesh rectopexy (n=20); laparoscopic posterior sutured rectopexy (n=20)	Females with complete rectal prolapse were treated with sutured/mesh rectopexy. The clinical endpoints were complete rectal prolapse recurrence rates and continence/constipation scores	The median operative durations were 77.21 minutes and 120.9 minutes in sutured rectopexy and mesh rectopexy, respectively. The rectal prolapse recurrence rates were higher in patients with sutured rectopexy, compared to those with mesh rectopexy (11.1% vs. 5.3%). Wexner Incontinence Scores statistically declined in both study groups, following the respective procedures; however, no pre- versus post-procedure differences were observed for Wexner Constipation Scores
Raftopoulos et al. [37]	2005	Retrospective study	643 patients	The Cox proportional hazards and chi-squared test approaches were used to evaluate rectal prolapse recurrence rates in patients with sutured versus mesh rectopexy	While the rectal prolapse recurrence rates did not statistically differ between the study groups, they were also not impacted by the rectopexy approach, access means, surgical technique, gender, and age of patients
Sahoo et al. [38]	2014	Retrospective study	Laparoscopic mesh rectopexy (n=38); laparoscopic suture rectopexy (n=32)	Patients were evaluated for postprocedural morbidity, rectal prolapse recurrence, constipation, fecal incontinence, surgery duration, hospital stay, and first bowel movement	Statistically significant improvements in constipation and continence were observed with suture rectopexy versus mesh rectopexy. No statistically significant differences in all other parameters were observed between the study groups
Usman et al. [39]	2023	Retrospective study	Laparoscopic suture rectopexy (n=34); laparoscopic mesh rectopexy (n=41)	Patients with rectal prolapse were evaluated for postprocedural constipation, incontinence, recurrence rates, wound-related complications, intraoperative bleeding, hospital stays, bowel activity, and operative duration	Compared to mesh rectopexy, suture rectopexy was associated with postoperative improvements in hospital stay duration, bowel activity, and operative times. Mesh rectopexy was associated with wound-related complications; however, suture rectopexy was devoid of intraoperative bleeding. Mesh rectopexy had higher recurrence rates, compared to suture rectopexy (9.8% vs. 2.9%)
Yehya et al. [40]	2020	A randomized, comparative, prospective study	Laparoscopic mesh rectopexy (n=32); laparoscopic suture rectopexy (n=32)	Each of the study groups was postoperatively evaluated for fecal incontinence, constipation, rectal prolapse recurrence rates, and operative time	The mesh rectopexy superseded the suture rectopexy due to better incontinence improvement (100% vs. 92.8%), reduced constipation incidence (3.3% vs. 3.57%), and lack of rectal prolapse recurrence (0% vs. 14.2%)
Bhandarwar et al. [41]	2017	Retrospective study	Laparoscopic mesh rectopexy (n=25); laparoscopic suture rectopexy (n=23)	Patients of the age group 25-75 years underwent laparoscopic suture or mesh rectopexy for complete rectal prolapse. While suture rectopexy utilized unabsorbable sutures, synthetic composite meshes were used in the mesh rectopexy group	The mean operative duration of the rectopexy patients was less than those who underwent suture rectopexy (96±10 vs. 124±10 minutes). Compared to mesh rectopexy, suture rectopexy resulted in a greater improvement in constipation (75% vs. 33%). While incontinence improvement did not differ between the study groups, suture rectopexy resulted in early improvements in bowel movements and postoperative flatus

Primary (postoperative) outcomes

Rectal Prolapse Recurrence

**Figure 2:**
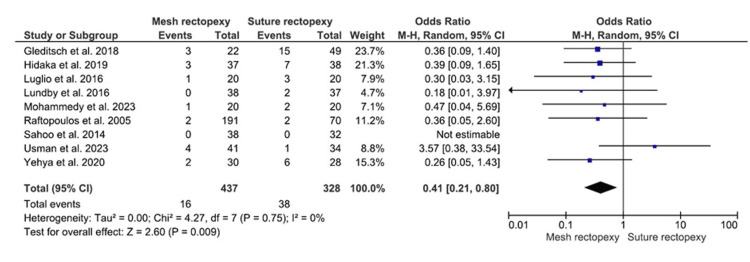
Rectal prolapse recurrence Studies by Gleditsch et al. [31], Hidaka et al. [32], Luglio et al. [34], Lundby et al. [35], Mohammedy et al. [36], Raftopoulos et al. [37], Sahoo et al. [38], Usman et al. [39], and Yehya et al. [40]

The rectal prolapse recurrence was evaluated in 437 patients with mesh rectopexy and 328 patients with suture rectopexy (Figure 2). Statistically significant lower odds for rectal prolapse recurrence were observed with mesh rectopexy, compared to suture rectopexy (OR: 0.41, 95% CI 0.21-0.80; p=0.009). Notably, low heterogeneity in the included studies was confirmed by 0% I2 value (p=0.75).

Constipation Improvement

**Figure 3:**
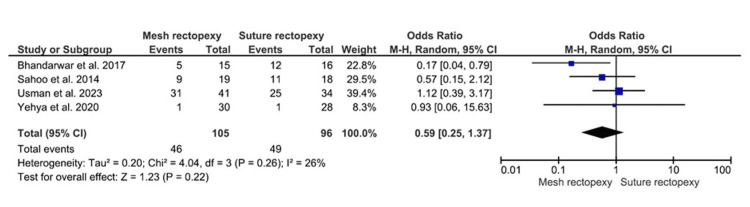
Constipation improvement Studies by Sahoo et al. [38], Usman et al. [39], Yehya et al. [40], and Bhandarwar et al. [41]

The constipation improvement occurrences were observed in 105 mesh rectopexy and 96 suture rectopexy patients. No statistically significant differences in postoperative constipation improvement were observed between mesh and suture rectopexy groups (OR: 0.59, 95% CI 0.25-1.37; p=0.22) (Figure 3). The overall low heterogeneity was further observed among the corresponding studies (I2=26%; p=0.26). 

Operative Time (Minutes)

**Figure 4:**
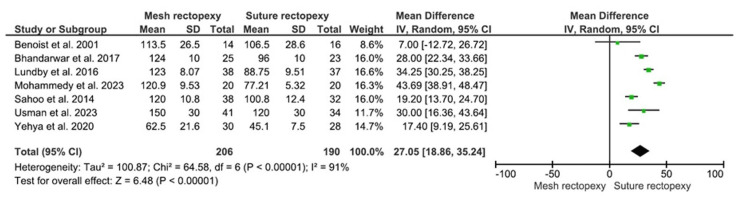
Operative time (minutes) Studies by Benoist et al. [33], Lundby et al. [35], Mohammedy et al. [36], Sahoo et al. [38], Usman et al. [39], Yehya et al. [40], and Bhandarwar et al. [41]

The operative duration was evaluated in 206 patients in the mesh rectopexy group and 190 patients in the suture rectopexy group. The longer mean operative time was reported with mesh rectopexy versus suture rectopexy (MD: 27.05, 95% CI 18.86-35.24; p<0.00001) (Figure 4). Of note, the respective studies had high heterogeneity (I2=91%; p<0.00001), increasing the risk of heterogeneity bias. 

Secondary (postoperative) outcomes

CCCS

**Figure 5:**
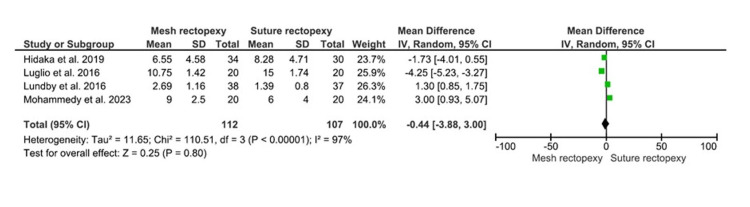
CCCS Studies by Hidaka et al. [32], Luglio et al. [34], Lundby et al. [35], and Mohammedy et al. [36] CCCS: Cleveland Clinic Constipation Score

The CCCS were compared between 112 mesh rectopexy patients and 107 suture rectopexy patients. Findings, however, revealed no statistically significant differences in CCCS between the study groups (MD: -0.44, 95% CI -3.88-3.00, p=0.80) (Figure 5). A high heterogeneity was observed among the included studies (I2=97%; p<0.00001).

CCIS

**Figure 6:**
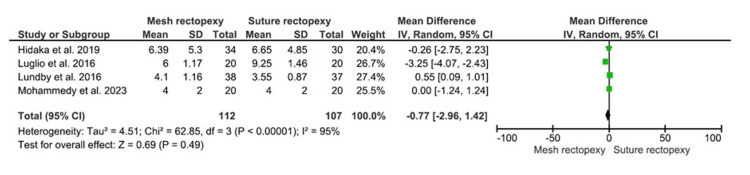
CCIS Studies by Hidaka et al. [32], Luglio et al. [34], Lundby et al. [35], and Mohammedy et al. [36] CCIS: Cleveland Clinic Incontinence Score

The CCIS were evaluated among 112 and 107 patients with mesh rectopexy and suture rectopexy, respectively (Figure 6). No statistically significant differences were observed in CCIS between mesh and suture rectopexy (MD: -0.77, 95% CI -2.96-1.42; p=0.49). Additionally, the included studies were found to have a high heterogeneity level (I2=95%; p<0.00001).

Intraoperative Bleeding

**Figure 7:**
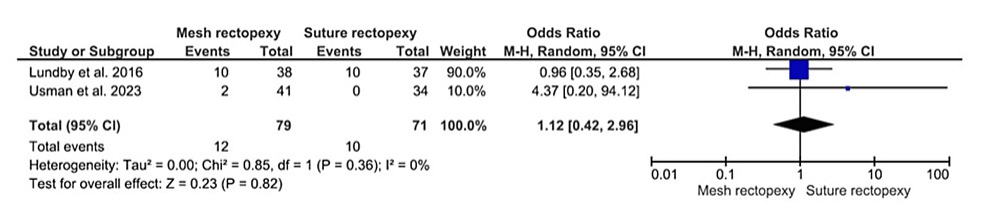
Intraoperative bleeding Studies by Lundby et al. [35] and Usman et al. [39]

Seventy-nine patients with mesh rectopexy and 71 patients with suture rectopexy were evaluated for intraoperative bleeding, which did not differ significantly between the study groups (OR: 1.12, 95% CI 0.42-2.96; p=0.82) (Figure 7). A low heterogeneity was, however, indicated by the 0% I2 value (p=0.36).

Hospital Stay Duration (Postoperative Days)

**Figure 8:**
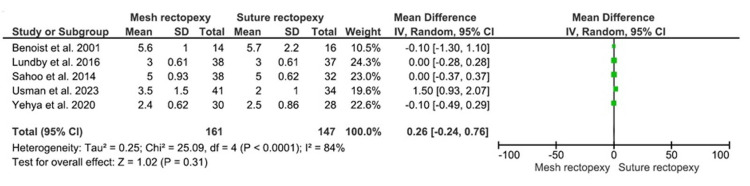
Hospital stay duration (postoperative days) Studies by Benoist et al. [33], Lundby et al. [35], Sahoo et al. [38], Usman et al. [39], and Yehya et al. [40]

The mean hospital stay duration was compared between 161 mesh rectopexy and 147 suture rectopexy patients and did not statistically differ between the study groups (MD: 0.26, 95% CI -0.24-0.76; p=0.31) (Figure 8). The I2 value (84%) indicated a high heterogeneity among the included studies (p<0.0001). 

Mortality

**Figure 9:**
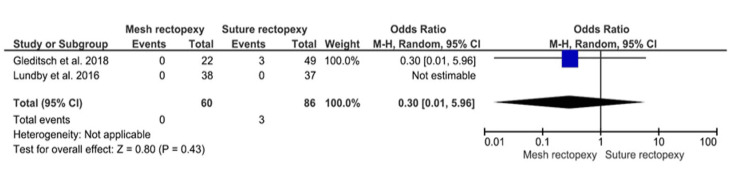
Mortality Studies by Gleditsch et al. [31] and Lundby et al. [35]

No statistically significant differences in postoperative all-cause mortality were observed between 60 mesh rectopexy patients and 86 suture rectopexy patients (OR: 0.30, 95% CI 0.01-5.96; p=0.43) (Figure 9). 

Overall Postoperative Complications

**Figure 10:**
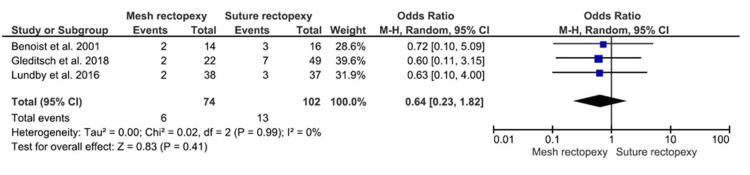
Overall postoperative complications Studies by Gleditsch et al. [31], Benoist et al. [33], and Lundby et al. [35]

The mesh rectopexy (n=74) and suture rectopexy (n=102) groups did not differ in terms of overall postoperative complications (OR: 0.64, 95% CI 0.23-1.82; p=0.41) (Figure 10). The heterogeneity across the respective studies was reportedly minimal (I2=0%; p=0.99). 

Surgical Site Infection

**Figure 11:**
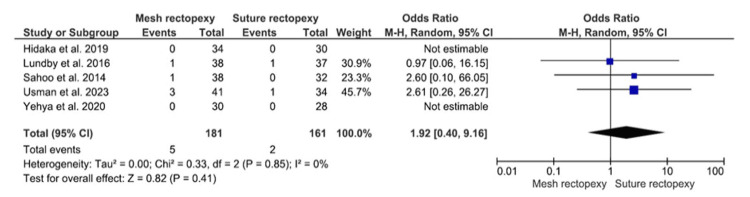
Surgical site infection Studies by Hidaka et al. [32], Lundby et al. [35], Sahoo et al. [38], Usman et al. [39], and Yehya et al. [40]

Surgical site infection was compared among 181 mesh rectopexy and 161 suture rectopexy participants (Figure 11). Findings revealed no statistically significant differences in surgical site infection between the study groups (OR: 1.92, 95% CI 0.40-9.16; p=0.41). A minimal heterogeneity was, however, reported among the included studies (I2=0%; p=0.85).

ROB

**Figure 12:**
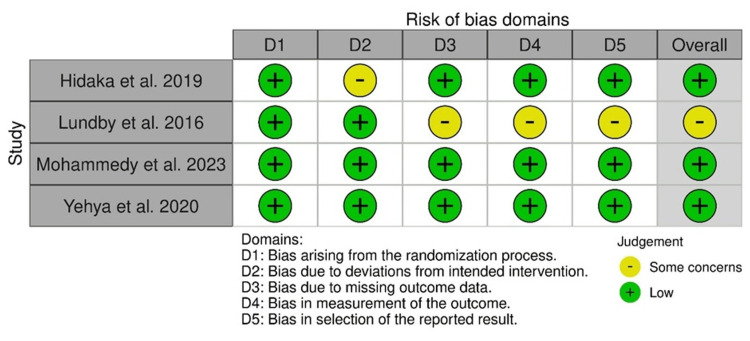
ROB-2: traffic light plot Studies by Hidaka et al. [32], Lundby et al. [35], Mohammedy et al. [36], and Yehya et al. [40]

The ROB assessment of four randomized controlled studies via the ROB-2 tool indicated the intervention deviation bias in the study by Hidaka et al., as well as missing outcome data-related bias, outcome measurement bias, and result selection bias in the study by Lundby et al. The overall results indicated a low ROB in the included studies (Figure 12).

**Figure 13:**
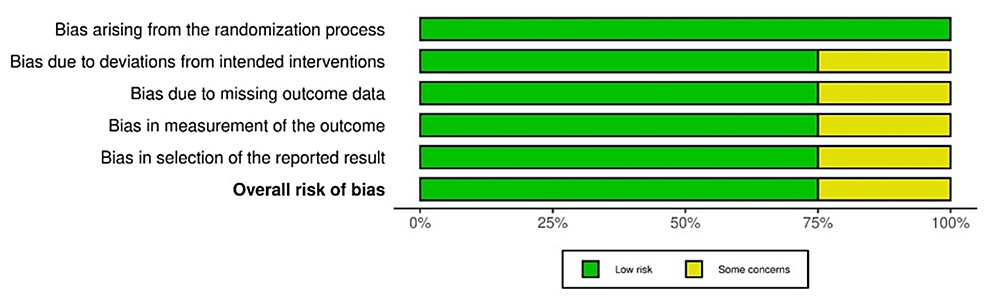
ROB-2: summary plot

Overall, low ROB was reported in 75% of studies, while some concerns were observed in 25% of studies, with randomized controlled/comparative design (Figure 13). 

**Figure 14:**
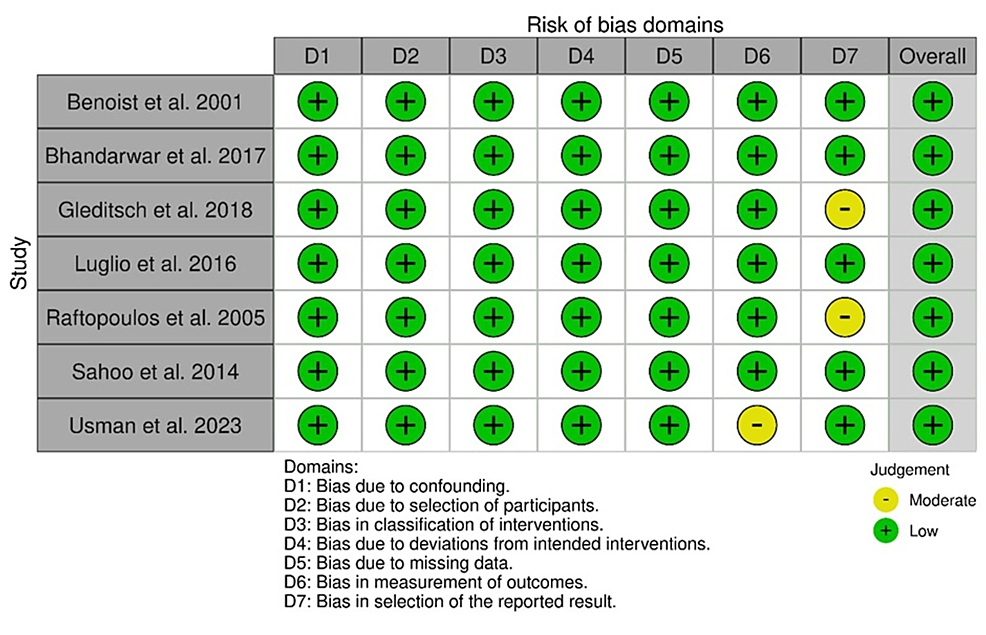
ROBINS-I: traffic light plot Studies by Gleditsch et al. [31], Benoist et al. [33], Luglio et al. [34], Raftopoulos et al. [37], Sahoo et al. [38], Usman et al. [39], and Bhandarwar et al. [41]

The statistical evaluation of 11 observational studies via the ROBINS-I method revealed an overall low ROB. However, a moderate bias in outcome measurement was observed in the study by Usman et al., and a moderate bias in reporting was observed in the studies by Gleditsch et al. and Raftopoulos et al., respectively (Figure 14).

**Figure 15:**
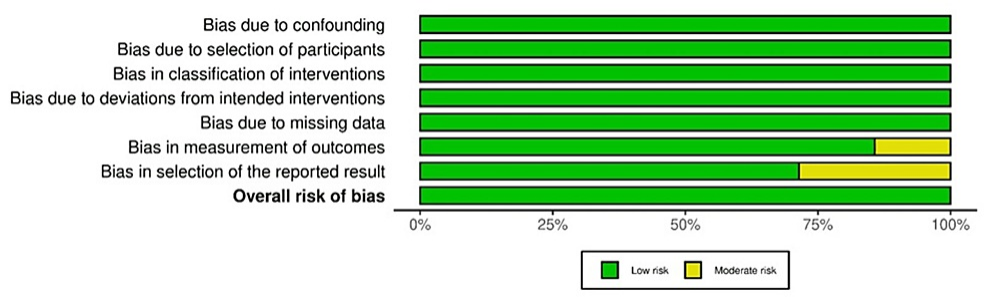
ROBINS-I: summary plot

Additionally, <10% and >90% of overall observational studies had moderate ROB and low ROB, respectively (Figure 15). 

Discussion

A statistically significant low recurrence of rectal prolapse and longer mean operative duration were observed in patients with mesh rectopexy, compared to suture rectopexy [31-41]. Of note, no significant differences between the study groups were observed in constipation improvement, CCCS, CCIS, intraoperative bleeding, hospital stay duration, mortality, overall postoperative complications, and surgical site infection.

The low rectal prolapse recurrence finding in the mesh rectopexy group in this study contradicted the finding from a recent prospective study by Schabl et al. indicating a high recurrence of rectal prolapse with mesh rectopexy, specifically in patients with a prior history of multiple surgeries. Notably, a recurrence rate of 3.7-15.4% is reported after mesh rectopexy, in patients without a past history of surgery for rectal prolapse [42]. However, following the abdominal approach, an 8% risk of a second recurrence is reported in the medical literature [43]. Contrastingly, outcomes of a systematic review by Lobb et al. and a retrospective study by Takahashi et al. reveal an elevated recurrence rate of rectal prolapse following suture rectopexy, when compared with ventral mesh rectopexy (7-15% vs. 5-8.8%) [11,17]. A recent meta-analysis by van der Schans et al. reveals a comparatively lower risk of rectal prolapse recurrence with biologic versus synthetic mesh rectopexy [44]. However, the associated risk factors of recurrence include CCCS (≧9), FISI score (>34), sacral fixation (tacks), length of external rectal prolapse (>4 cm), past rectal prolapse surgery, ASA physical status 3, body mass index (≧22), and age (>80 years) [45]. 

The finding of the current study regarding the longer operative time with mesh rectopexy, compared to suture rectopexy, concords with the outcome of the systematic review by Hajibandeh et al. [18]. Contrarily, the meta-analysis results of Emile et al. defy statistically significant differences in operative time between suture and mesh rectopexy [19]. However, findings from a retrospective study by Sahoo et al. also specify that mesh rectopexy requires 120±10.8 minutes, compared to 100.8±12.4 minutes for suture rectopexy [38]. The increased procedural time in mesh rectopexy is due to the additional workup warranted for circumferential rectal mobilization and additional dissection [46]. Our results, however, matched the meta-analysis outcomes of Hajibandeh et al. that indicated similarities in hospital stay duration, surgical site infection, CCCS, and CCIS between suture rectopexy and mesh rectopexy [18].

The laparoscopic ventral mesh rectopexy specifically aims to restore the function as well as the anatomy of the prolapsed rectum [47]. However, postoperative hemorrhoid development, fistula due to intrarectal mesh migration, and mesh-related erosion are the potential complications contributing to the recurrence of rectal prolapse after mesh rectopexy [48,49]. It is therefore important to periodically evaluate the functional outcomes of mesh rectopexy to determine its impact on the health-related quality of life and recovery paradigm. Of note, most patients treated with mesh rectopexy experience a noticeable improvement in Birmingham Bowel and Urinary Symptom (BBUS) score, ODS score, Wexner Fecal Incontinence (WFI) score, bladder dysfunction, and incontinence [47]. That is why despite a low response rate, mesh rectopexy remains a gold standard for treating complete rectal prolapse.

The laparoscopic suture rectopexy is a conservative approach for attaching the sacrum to the mesorectum via sutures [50,51]. However, the fragility of the mesorectum leads to poor rectal fixation that increases the risk of rectal prolapse recurrence in the treated patients. The suturing of the sacrum with the rectal wall's seromuscular layer is performed due to tension-related lengthening of the mesorectum [17]. While the mucosal layer's penetration of rectal sutures increases the risk of infection, the erosion of the rectal wall at the suture location triggers the onset of perirectal abscess. Additionally, postoperative spondylitis and intra-abdominal abscess are the potential complications of suture rectopexy, requiring long-term management [17,52]. Consequentially, the inflammation of the appendix after mesh rectopexy warrants management with ileostomy covering, appendectomy, and mesh excision [53]. Similarly, ileostomy covering and anterior resection are the required treatment approaches for patients who develop rectal mesh fistulation, after mesh rectopexy [17]. Future randomized controlled studies should specifically evaluate and compare these mesh versus suture rectopexy complications to improve the personalized management of complete rectal prolapse. 

Limitations

Findings from this systematic review and meta-analysis cannot be generalized for all patients with complete rectal prolapse due to several limitations, including high heterogeneity, a small sample size, and a smaller number of randomized controlled studies. Additionally, we could not perform a subgroup analysis of the primary and secondary endpoints based on the demographic characteristics, including prior surgeries, comorbidities, body mass index, ASA grade, age, gender, preoperative complications, and previous treatments. This limitation could further impact the reliability of our results. 

## Conclusions

This study revealed a longer operative duration due to the different levels of skills of surgeons in various studies and the low postoperative rectal prolapse recurrence in patients with laparoscopic mesh rectopexy versus those with laparoscopic suture rectopexy. However, no differences between the groups were reported for constipation improvement and secondary endpoints. Future studies should further examine the potential causes and outcomes of rectal/mesh rectopexy to improve the decision-making for the surgical management of full-thickness rectal prolapse.
